# Explainable artificial intelligence for searching frequency characteristics in Parkinson’s disease tremor

**DOI:** 10.1038/s41598-023-45802-z

**Published:** 2023-10-30

**Authors:** Rui En Lee, Ping Yi Chan

**Affiliations:** School of Engineering, Monash University Malaysia, Bandar Sunway, Subang Jaya, Selangor Malaysia

**Keywords:** Diagnostic markers, Motor control, Neurophysiology, Biomedical engineering

## Abstract

The distinction between Parkinson’s disease (PD) and essential tremor (ET) tremors is subtle, posing challenges in differentiation. To accurately classify the PD and ET, BiLSTM-based recurrent neural networks are employed to classify between normal patients (N), PD patients, and ET patients using accelerometry data on their lower arm (L), hand (H), and upper arm (U) as inputs. The trained recurrent neural network (RNN) has reached 80% accuracy. The neural network is analyzed using layer-wise relevance propagation (LRP) to understand the internal workings of the neural network. A novel explainable AI method, called LRP-based approximate linear weights (ALW), is introduced to identify the similarities in relevance when assigning the class scores in the neural network. The ALW functions as a 2D kernel that linearly transforms the input data directly into the class scores, which significantly reduces the complexity of analyzing the neural network. This new classification method reconstructs the neural network’s original function, achieving a 73% PD and ET tremor classification accuracy. By analyzing the ALWs, the correlation between each input and the class can also be determined. Then, the differentiating features can be subsequently identified. Since the input is preprocessed using short-time Fourier transform (STFT), the differences between the magnitude of tremor frequencies ranging from 3 to 30 Hz in the mean N, PD, and ET subjects are successfully identified. Aside from matching the current medical knowledge on frequency content in the tremors, the differentiating features also provide insights about frequency contents in the tremors in other frequency bands and body parts.

## Introduction

Significant clinical overlaps exist between Parkinson’s disease (PD) and essential tremor (ET), which poses a threat in the medical field when diagnosing a patient with these diseases. As a result, 25% of PD patients are misdiagnosed as ET^1^, which causes some significant loss of medicinal resources since the interventions for these diseases differ considerably^2^. Besides, the diagnosis of these diseases has to be done accurately and early for a successful mitigation of the diseases.

Machine learning techniques such as K-nearest neighbour (KNN), Naive Bayes, support vector machine (SVM), random forest, decision tree, and ensemble learning are the most popular techniques used in the classification of tremors. For instance, using accelerometry on hand as a dataset, Skramangkas et al.^3^ has tried the aforementioned classifiers. They have achieved up to 100% accuracy using the quadratic SVM, cubic SVM, and linear SVM and concluded that SVM is the best classifier. However, extensive data preprocessing and feature extraction have to be done before executing these machine-learning techniques.

Recently, neural networks have been a popular option in classifying PD, ET, and normal patients(N). Unlike machine learning techniques, minimal feature extraction has to be done before executing the neural networks, which allows the neural network to explore and search for new, novel features in a wider space that is not limited by the predetermined feature extraction, which is, most of the time, based on the prior knowledge on the domain. Relying on the automatic feature extraction benefit of neural networks, Xing et al.^4^ did minimal data preprocessing to create inputs to train the convolutional neural network (CNN) for differentiating between PD and ET and has reached 78% accuracy. Arvind et al.^5^ have used a recurrent neural network (RNN) to detect PD in rest tremors with an accuracy of 95.6%. On the other hand, a convolutional long short-term memory (LSTM) network is also used by Oktay^6^ to reach 90% accuracy in differentiating between PD and ET.

Despite achieving high accuracy, almost all of the previous machine learning-based literatures did not understand the algorithms trained to extract differential characteristics between PD and ET. The algorithms are often treated as a black box model that converts the input into an output that classifies the data. The means of how multilayer neural networks differentiate the data can be interpreted by using explainable artificial intelligence (explainable AI), which is a method to explain the internal workings and logics of the trained neural networks. Thus far, only one work reported the use of explainable AI in PD and ET tremor differentiation. Shahtalebi et al.^7^ developed an AI algorithm that achieved 95.6% accuracy and further developed an explainable AI, Gradient-weighted class Activation Mapping, to understand the AI model. From the efforts to explain the AI model, they reported that the PD is characterised by the low-frequency vibration on hand, and ET is mainly characterised by the high spectral activity.

Since the architecture of the neural network is heavily complex, conventional explainable AI methods might not be suitable for this application. Bach et al.^8^ developed layer-wise relevance propagation (LRP) to explain the decision-making of a convolutional neural network for each parameter of the input data. Then, Becker et al.^9^ and Arras et al.^10^ extended the application of LRP to LSTM layers in recurrent neural networks to classify audio signals and sentimental analysis, respectively. Since the tremor signal is also a time-series data, which requires the use of a recurrent neural network, LRP will be the primary method that will be used for explaining the neural network in this work. By inspecting the similarities between the relevance of each parameter for data of each class, differentiating characteristics of class N, PD, and ET can be formulated.

In this paper, a novel method for further simplifying the outcome of the LRP in explaining the neural networks, approximate linear weights (ALW), is proposed. With the ALW that approximates the relationship between inputs and class scores, differentiating features between tremors of PD and ET in terms of the relative magnitude of frequencies from 3 Hz to 30 Hz are identified. The focus of our study is on presenting the ALW, which gives a clue about the differentiating features of the three classes, while the performance of classification using the ALW is a side outcome. The proposed ALW improves the interpretability of the relationship between the inputs and class scores.

## Methodology

**Figure 1:**
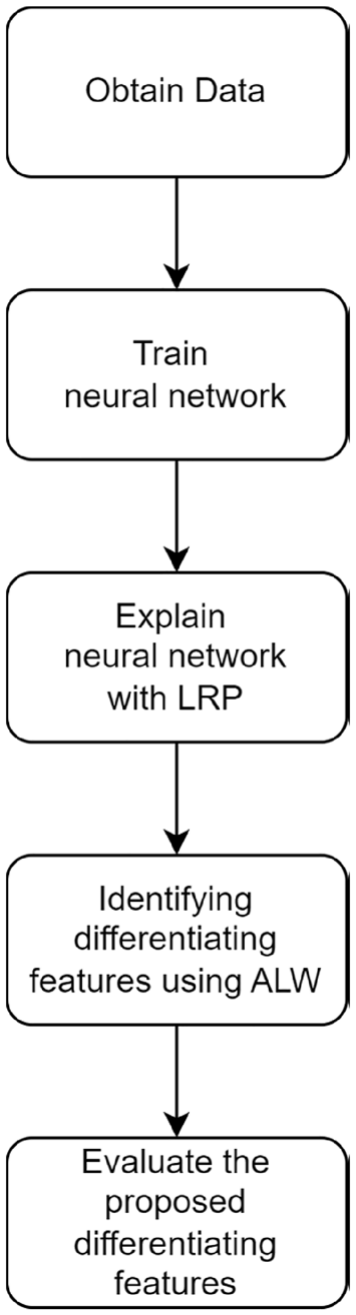
Methodology flowchart.

The methodology is summarized in Fig. 1. After obtaining data, a neural network is trained to classify the datasets. Then, the neural network is explained using explainable AI techniques. Based on the findings, differentiating characteristics between PD, ET and N are formulated.

The Medical Research Ethics Committee, Secretariat of National Institutes of Health, Malaysia, approved the clinical study (protocol no. NMRR-14-1694-21740 [IIR]) carried out mainly in the Neurology Clinic of Penang General Hospital. The research protocol was designed in accordance with the National Institutes of Health guidelines. All subjects were recruited following written informed consent.

Tremors were measured with attitude and headings reference system model SBG IG–500A (SBG Systems, Rueil–Malmaison, France) on the lower arm (L), hand (H), and upper arm (U), when the upper limb was in this neutral position. The quaternion data from the system were acquired and analysed using LabVIEW™ software (National Instruments Corporation, Austin, Texas)^11^.

The upper limb resting (REST) and outstretching postures (OUT) were performed according to the protocols in MDS–UPDRS upon obtaining permission from the International Parkinson and Movement Disorder Society. The wing posture (WING), drinking action (DRINK), and finger-nose-finger (FNF) were carried out according to the protocol of the Washington Height–Inwood Genetic Study of Essential Tremor Tremor Rating Scale (wTRS).

This study processes the tremors of a total of 67 N subjects, 87 PD subjects, and 18 ET subjects, whose average ages are 63.62 ± 11.67. The resulting sample size for each action and subject group are shown in Table 1.

**Table 1:** Sample size of the tremor signal dataset.

Action	All Actions	DRINK	FNF	OUT	REST	WING	EAT
N	750	126	125	124	126	125	124
PD	667	118	116	127	131	108	77
ET	153	25	28	29	30	25	16
Total	1570	269	269	280	287	258	217

The data recordings are interpolated using Piecewise Cubic Hermite Interpolating Polynomial (PCHIP) for a uniform timestep. The data is then filtered with a bandpass filter with a cut-off frequency of 3 Hz to 30 Hz and preprocessed into Short Time Fourier Transform (STFT) based input parameters. The data are of different lengths, particularly due to the varying reaction times of subjects and the removal of irrelevant motions such as coughing. All the data with extra timesteps are removed until all data are of the same length as the shortest data. After preprocessing, the time resolution is at 4.7 ms while the frequency resolution is at 0.7825 Hz, with the frequency starting at 3.12 Hz and ending at 29.69 Hz.

**Figure 2:**
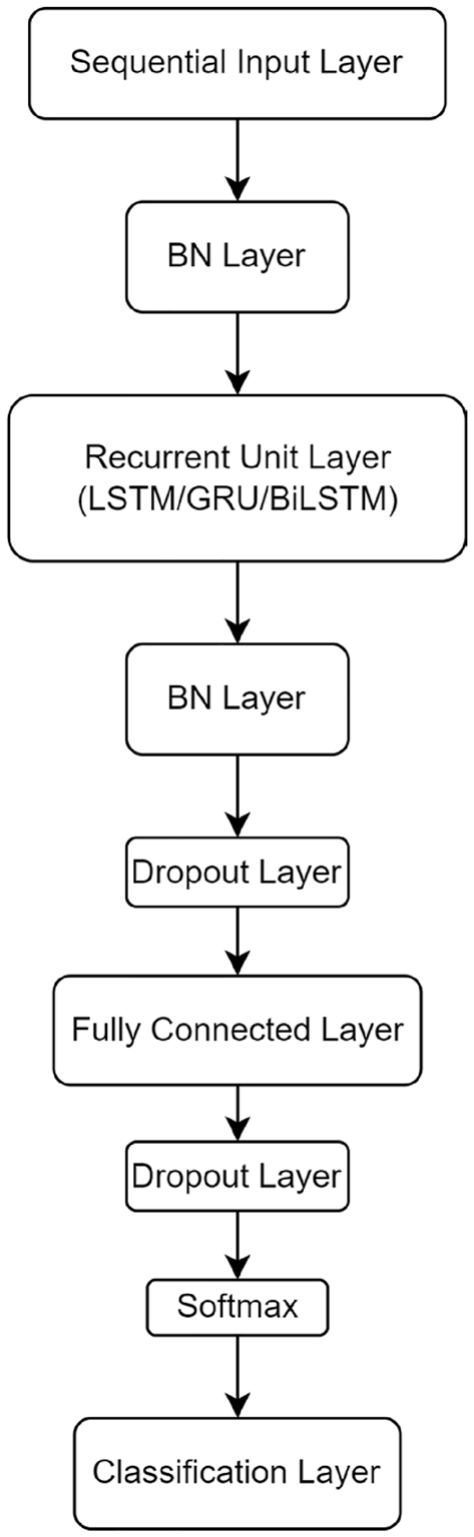
BiLSTM-based neural network architecture.

The neural network used is a bidirectional long-term short memory (BiLSTM) based recurrent neural network. The architecture of the neural network is shown in Fig. 2.

While training the neural network, it is a common practice to split the dataset into a training set, validation set and testing set. The training set is the input fed into the neural network, while the validation set is the intermediate testing set used to ensure the correctness of training of the neural network. The testing set is the isolated dataset that the neural network has not seen before, and thus it is used to accurately evaluate the neural network after training and validation. A class-balanced 30 dataset is isolated to serve as the testing dataset. The rest are divided into training and validation data, with a training-validation split of 80:20 for the remaining dataset. In addition, the batch normalization (BN) layer is used in between the recurrent units and fully connected layers to standardize the inputs using the self-learned means and standard deviations. Dropout layers with 40% dropout probability are used to prevent overfitting.

To understand how the features are formulated, LRP can be applied on each individual data classification to obtain insight on the decision-making in the neural network. LRP provides relevance of each input value on the prediction made by the neural network. The LRP implemented is based on Arras et al.^10^. For each data, LRP is executed for each class, with the class scores outputted by the softmax layer used as the relevance at the final layer, as the class scores correspond to the probability of the data being assigned into the class. The rule of LRP differs at each layer, depending on the type and output mode of the layer. However, the propagation of the relevance generally follows the rule of LRP through a linear layer shown as Eq. 1.

1 $$ R_{i} = \mathop \sum \limits_{j = 1}^{{N_{j} }} \frac{{w_{ij} z_{i} + \frac{{\delta b_{j} + \varepsilon sign\left( {z_{j} } \right)}}{{N_{i} }}}}{{z_{j} + \varepsilon sign\left( {z_{j} } \right)}}R_{j} $$

where $R_{i}$ is the relevance of input neuron, $R_{j}$ is the relevance of the output neuron, $z_{i}$ is input neuron, $z_{j}$ is the output neuron, the $b$ is the bias for output neuron $j$, $w_{ij}$ is the weight relating neuron $i$ and $j$, and $N_{j}$ is the total number of output neurons. The hyperparameters used are $\delta = 0.0$ and $\varepsilon = 0.001$.

In this project, a novel method, called Approximate Linear Weights (ALW) is proposed to approximate the relationship between inputs and class scores. The ALW approximates the neural network into linear weights as a 2D convolutional layer using the relevance obtained from LRP. In this way, the input-class scores relation can be explained with ease as compared to analyzing the entire neural network. A positive weight can be explained as a positive correlation between the input and the class score; thus, the input value is generally higher in that specific class. Similarly, a negative weight denotes a negative correlation between the input and the class score, thus data from that class will generally have a lower input value. The ALW introduced in this work, W can be calculated by computing the element-wise division of relevance of the input, $R$ by the input, $X$, followed by averaging across all data, as shown in Eq. 2. The D is the total number of data available in the dataset, c is the index of the class, and d is the index of each data. $R_{c, d}$ refers to the relevance of class $c$ for the $d$-th data.

2 $$ W_{c} = \frac{1}{D}\mathop \sum \limits_{d = 1}^{D} R_{ c, d} { \oslash }X_{d} $$

**Figure 3:**
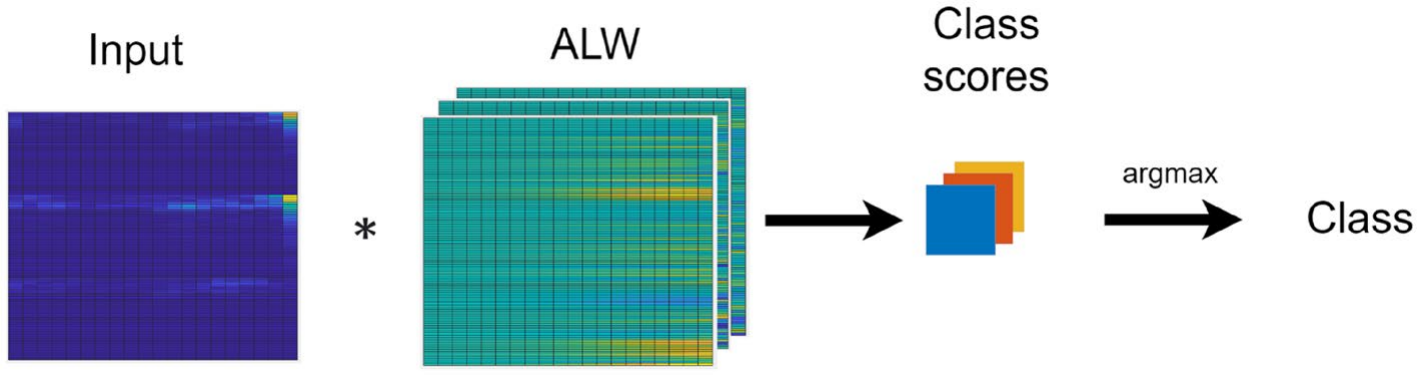
Visualization of ALW classification operation.

W is a kernel with dimensions $n \times \tau \times C$, where n is the input size, C is the number of classes. τ is a manipulatable variable, which is the number of timesteps of interest. Since the time-series data used in this project has a varying number of timesteps, T has to be selected to retain most of the information in the input. If the input has a number of timesteps greater than τ, only the first τ timesteps will be used to calculate W. The value of τ must be determined experimentally. The weights can also be used to calculate the class scores using Eqs. 3 and 4, which is visualized in Fig. 3.

3 $$ S = W*X_{{t \in \left[ {1, \tau } \right]}} $$

4 $$ \underline {c} = argmax\left( S \right) $$

where $\underline {c}$ is the predicted class.

In this project, $\tau$ = 20 is used. Two separate weights are taken to fit the neural network better $W_{start}$, which are the weights at the first $\tau$ timesteps, and $W_{end}$, which are the weights at the last $\tau$ timesteps. Equation 7 and 8 shows the updated formula to calculate the ALWs.

5 $$ W_{ ntc}^{start} = \frac{1}{D}\mathop \sum \limits_{d = 1}^{D} R_{{cd, t \in \left[ {1, \tau } \right]}} { \oslash }X_{{d, t \in \left[ {1, \tau } \right]}} $$

6 $$ W_{ntc}^{end} = \frac{1}{D}\mathop \sum \limits_{d = 1}^{D} R_{{cd, t \in \left[ {T - \tau + 1, T} \right]}} { \oslash }X_{{d, t \in \left[ {T - \tau + 1, T} \right]}} $$

**Figure 4:**
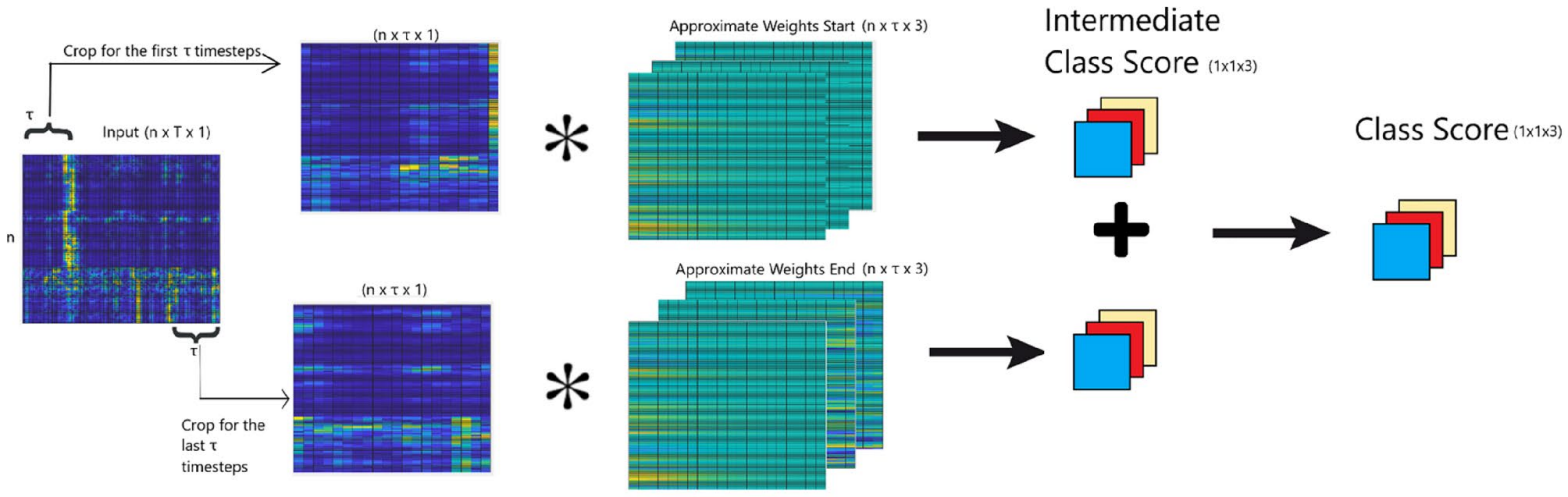
Visualisation of ALW classification operation.

Equations 7 and 8 shows the formula to calculate the predicted class using the new weights, which is the expanded version of Eqs. 3 and 4. Figure 4 shows the visualization of the classification operation. Since this algorithm has directly transformed the input into the class scores, the use of shallow classifiers is not required.

7 $$ S = W^{start} *X_{{d, t \in \left[ {1, \tau } \right]}} + W^{end} *X_{{t \in \left[ {T - \tau + 1, T} \right]}} $$

8 $$ \underline {c} = argmax\left( S \right) $$

Using the ALWs, direct, the neural network is simplified and is more interpretable, and linear relationships can be formulated between the inputs and class scores. Since the timesteps are also factored in the ALWs, affinity between the input and the class score over time can be observed. Thus, differentiating characteristics can be identified.

**Figure 5:**
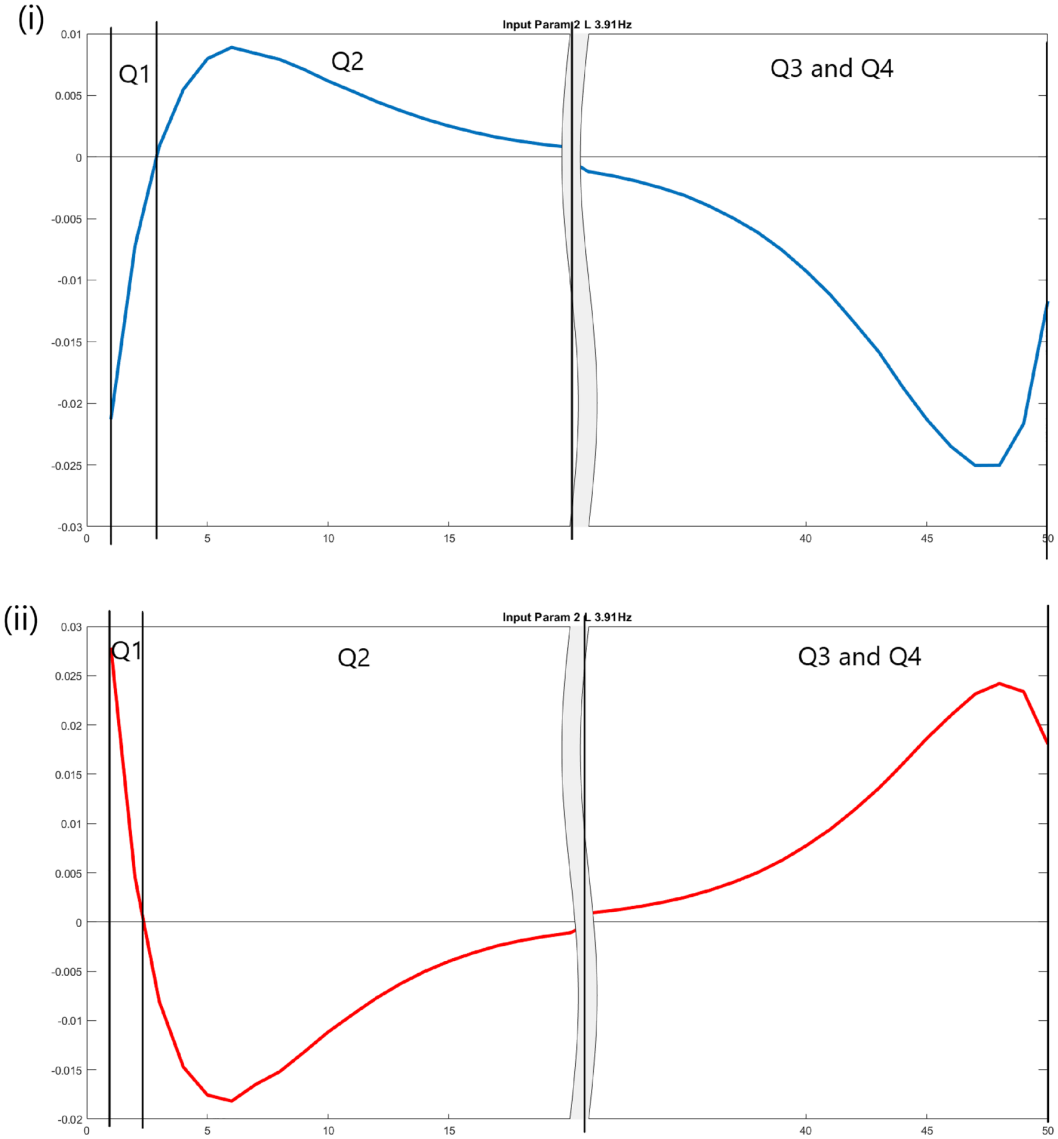
Examples of splitting the approximate weights into quarters. (i) Approximate weights of input parameter 2 for class N, which corresponds to 3.91 Hz at the lower arm. The quartered signs are NPNN. (ii) Approximate weights of input parameter 2 for class PD, which corresponds to 3.91 Hz at the lower arm. The quartered signs are PNPP.

To group the ALWs, the timesteps is divided into 4 quarters: the first $W_{start}$ timestep up until a change in sign in $W_{start}$, $W_{start}$ after the change in sign, $W_{end}$ before a change in sign, $W_{end}$ after the change in sign. If there are no sign change at $W_{start}$, the second quarter will follow the sign of the first quarter. If there are no sign change at $W_{start}$, the fourth quarter will follow the sign of the third quarter. Figure 5 shows examples of splitting the approximate weights into quarters.

## Results and discussion

### Layer-wise relevance propagation (LRP)

**Figure 6:**
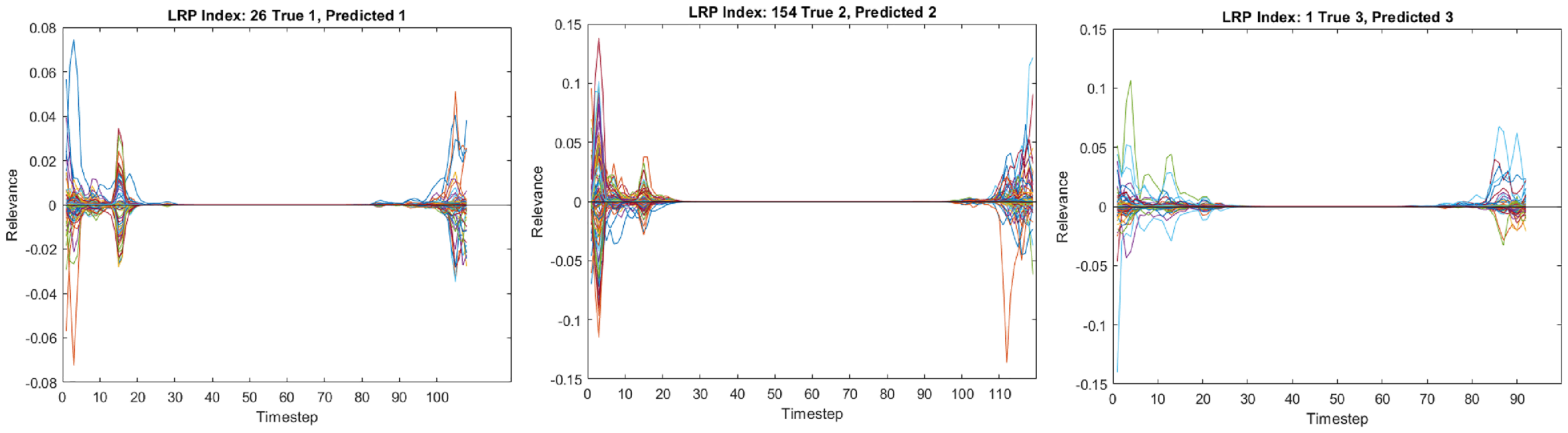
Plots of relevance against timesteps for each sample subject from class 1 (N), class 2 (PD), and class 3 (ET). Different lines refer to the relevance of each of the 105 sets of input parameters.

**Figure 7:**
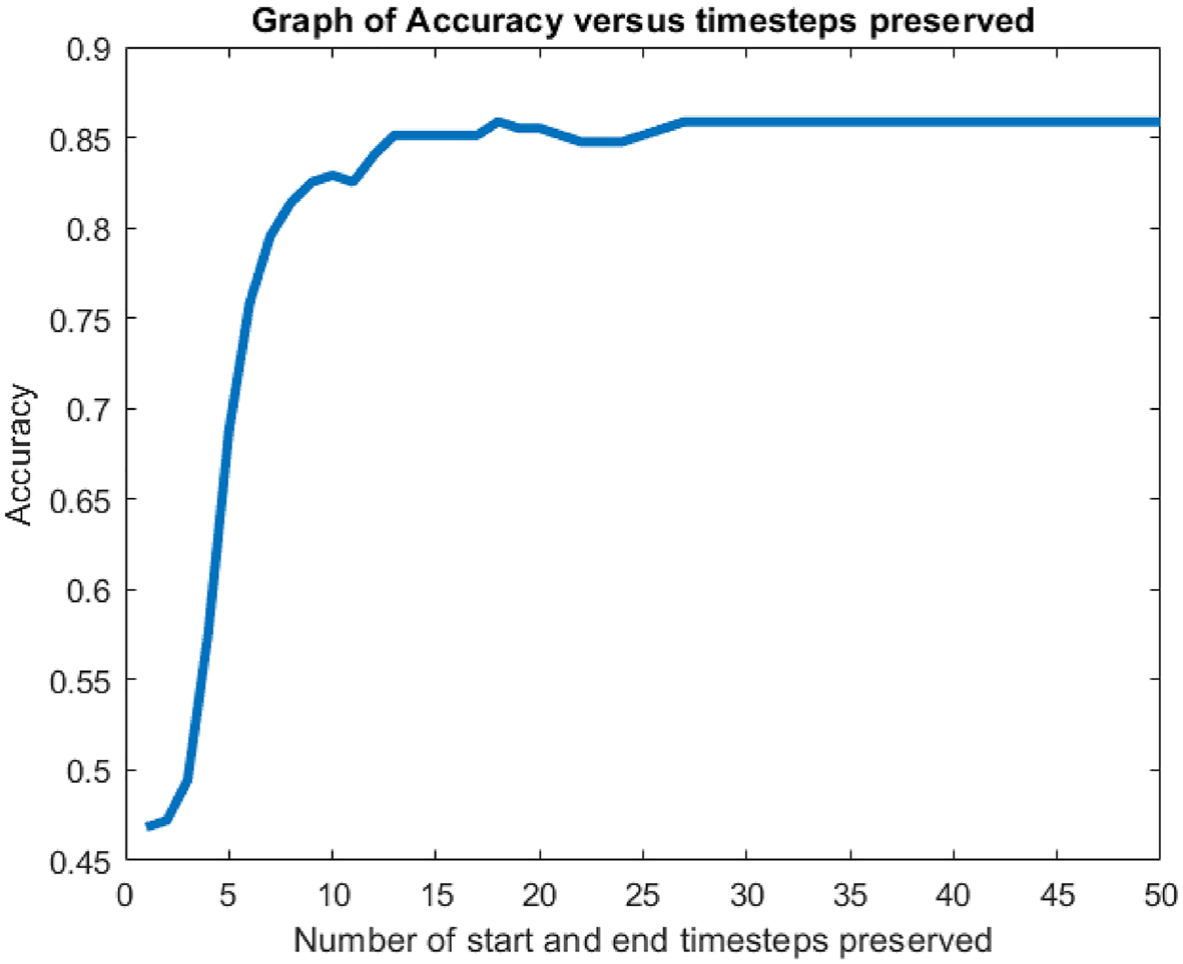
Graph of accuracy vs number of start and end timesteps preserved.

Results of LRP of three different data from each class for drinking action are shown in Fig. 6. Each timestep has 105 sets of corresponding input parameters, which contain information of the frequency and amplitude of the STFT. Each presented relevance value corresponds to a STFT input data. The relevance values of all inputs show that only the starting and the ending timesteps have significant contributions to the predicting mechanism. The middle timesteps have almost 0 relevance when there are more timesteps in the data. This is caused by the usage of biLSTM layers, whose forward LSTM unit is sensitive to the ending timesteps while the backward LSTM unit is sensitive to the starting timesteps. Information in the middle timesteps are discarded by the forget gate in the LSTM units. The significance of the starting and ending timesteps are proven in Fig. 7, since the neural network can achieve full classification at 20 preserved timesteps based on the time index. As shown in Fig. 7, The accuracy drops when using less than 20 preserved timesteps, and stays relatively constant when using more than 20 preserved timesteps. Therefore, it is concluded that only the first and last 20 timesteps are important for the neural network to classify correctly.

**Figure 8:**
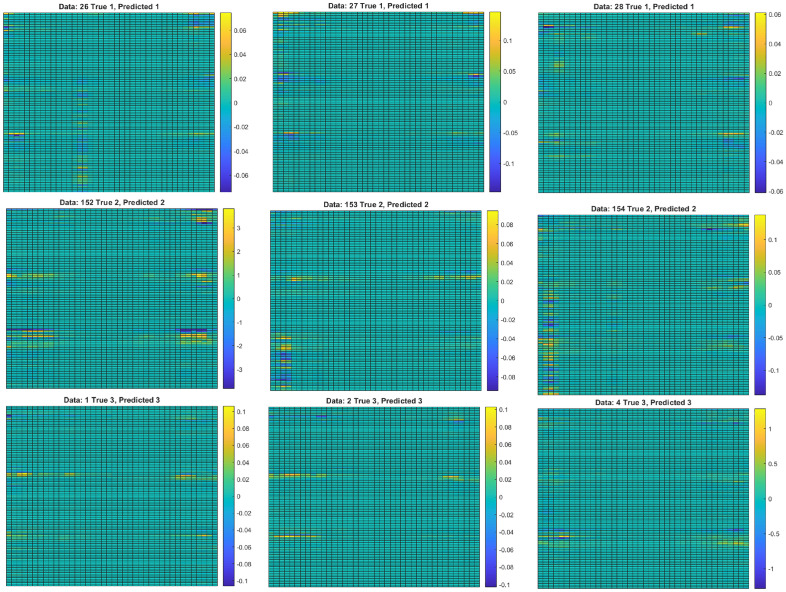
Heatmaps of relevance from class 1 (N), class 2 (PD), and class 3 (ET). The x-axis corresponds to the timesteps, and the y-axis corresponds to the frequency in top-down ascending order from 3.1 Hz to 28.9 Hz, stacked for the lower arm, hand, and upper arm. Only the first and last 20 timesteps are plotted since the middle timesteps have relevances of close to 0.

For better visibility, heatmaps are also plotted using MATLAB R2021b^12^ for the first and last 20 timesteps in Fig. 8. The x-axis is the timestep in 4.7 ms interval, and y-axis is the frequency ranging from 3.12 to 29.69 Hz with 0.7825 Hz intervals (35 frequency steps), for the lower arm, hand and upper arm (a total of 105 input parameters). The colour of the heatmap indicates the relevance corresponds to the STFT input parameters as a whole. It is obvious that the parameters with the highest relevance are not the same across all data.

Though the relevance of the heatmaps in Fig. 8 highlights the important regions used in the classification by the neural network, it is challenging to delineate the distinction among the classes through observation. Thus, ALW is proposed to simplify the LRP yet captures the key distinct characteristics. The distinctions of the three classes are presented in terms of frequency domain in the ‘Differentiating Features Identification’ section.

**Figure 9:**
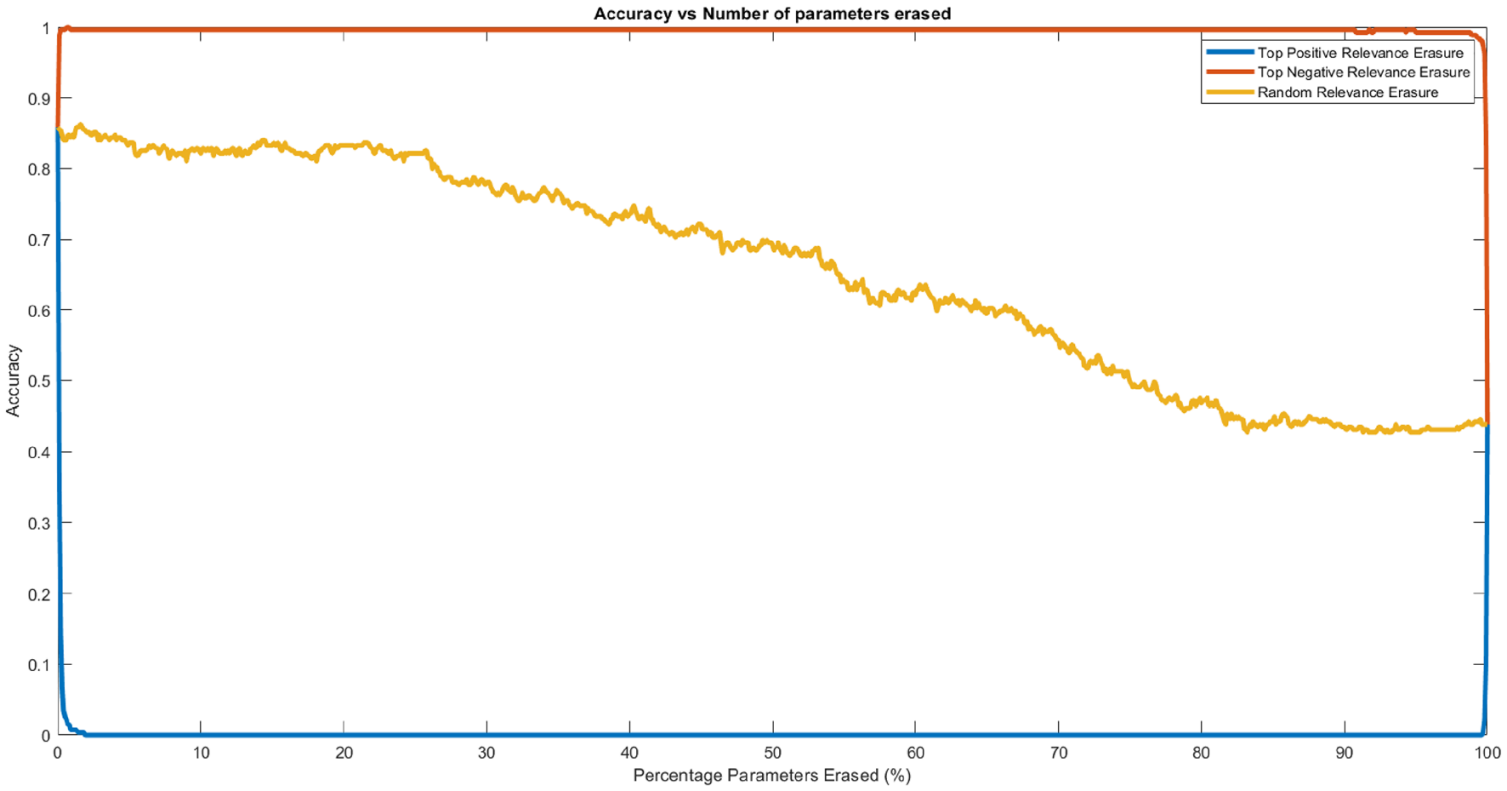
Graph of accuracies against the proportion of input parameters zeroed.

In order to verify the reliability of the LRP algorithm implemented, erasure analysis is used by zeroing individual input parameters at certain timesteps. If the input values with positive relevance are erased, the data should be incorrectly classified, while if input values with negative relevance are erased, the data should be correctly classified. Figure 9 shows the results of the erasure analysis. It is obvious that as positive relevant parameters care removed, the accuracies drop to almost zeros. Conversely, as negative relevant parameters are removed, the accuracy rises to 100%. The random erasure line serves as the control set to contrast the properties of the other two lines. At the end of the plots, all three lines converge to the same point since all parameters are removed and set to 0, and the dataset is the same for all three cases. This supports the hypothesis, and thus, the LRP implementation is correct.

It is difficult to identify any similarities between the relevance of data from the same class in Fig. 8. A novel method, known as ALW, can be used to generalise and find similarities between the relevance plots. Due to the usage of the biLSTM layers, it is observed that the relevance at the start and the end of the data is generally higher due to the forgetting properties of an LSTM unit that prioritises the more recent data (as explained in the first paragraph of this section). There are two spikes in the relevance, once at the start and once at the end. The high relevance at the start is caused by the backward LSTM unit, while the high relevance at the end is caused by the forward LSTM unit. Therefore, due to the high relevance observed at the beginning and at the end of the data, ALWs are calculated based on Eqs. 7 and 8, instead of Eq. 2.

The ALWs based on the neural network trained on input parameters derived from STFT simplified and drinking action is attached in the appendix. Some of the general signs of one class are unique to the other classes. For example, for a frequency of 3.12 Hz at the lower arm, ALW for N is positive, while the ALW for PD and ET are negative. This describes differentiating features for N since it stands out from the other classes.

Tables 2 and 3 shows the performance metrics using the original neural network and ALWs. The neural network has achieved 0.6–1.0 accuracy and an F1-score of 0.7–0.9, while the ALWs have achieved 0.5–0.9 accuracy and an F1-score of 0.5–0.9. The performance has degraded using the ALWs. This is because the weights only capture the linear aspects of the neural network. Non-linear aspects in the neural network, such as activation layers are neglected. Besides, the LRP implemented has some leakages of relevance due to the bias terms in the fully connected layer and the biLSTM layers and the forget gate in the LSTM layers. Since the performance metrics have only worsened slightly, it is concluded that the ALWs are a good linear approximation of the mechanism of the neural network. This method has traded off performance for interpretable purposes.

**Table 2:** Performance metrics of the original neural network.

Performance Metrics	All Classes	Class 1 (N)	Class 2 (PD)	Class 3 (ET)
Accuracy	0.8000	1.0000	0.8000	0.6000
F1	–	0.9523	0.7272	0.7059
Sensitivity	–	1.0000	0.8000	0.6000
Specificity	–	0.9500	0.8000	0.9500

**Table 3:** Performance metrics of the ALWs.

Performance Metrics	All Classes	Class 1 (N)	Class 2 (PD)	Class 3 (ET)
Accuracy	0.7333	0.9000	0.8000	0.5000
F1	–	0.8571	0.7273	0.5882
Sensitivity	–	0.9000	0.8000	0.5000
Specificity	–	0.9000	0.8000	0.9000

In our scope of study, the focus is on presenting the ALW and the clue on differentiating features, while the results of the ALW are the side outcome of the work.

### Differentiating features identification

Using the ALW with STFT as the input features, it is possible to identify the frequency contents that correlate with N, PD or ET. A summary of the sign of the weights is shown in Table 4, with the timesteps divided into quarters as described in Fig. 5. A positive weight denotes that the frequency should have a high magnitude for that class, whereas a negative weight denotes that the frequency should have a low magnitude. Some of the weights of an input feature are consistently positive or negative at all timesteps, which suggests the presence or absence of a dominant tremor frequency in that class. However, the weights of input features change their signs over time. For example, for frequency content 3.9 Hz at the lower arm in N, the weights change from negative to positive in the first half of the timesteps.

**Table 4:** Summary of correlation between tremor frequency at the lower arm, hand or upper arm and class N, PD, and ET.

Part	f (Hz)	Correlation														Correlation														Correlation											
		N				PD				ET				P	f (Hz)	N				PD				ET				P	f (Hz)	N				PD				ET			
L	3.1	P	P	P	P	N	N	N	N	N	N	P	P	H	3.1	P	N	N	N	N	P	N	N	N	N	P	P	U	3.1	P	P	P	P	N	N	N	N	N	N	N	N
L	3.9	N	P	N	N	P	N	P	P	N	N	P	P	H	3.9	P	P	P	P	N	N	N	N	P	N	P	P	U	3.9	N	N	P	P	P	P	N	N	N	P	P	P
L	4.7	P	N	N	N	N	P	P	P	P	P	N	N	H	4.7	N	N	N	N	P	P	P	P	N	P	N	N	U	4.7	N	N	P	P	P	P	N	N	N	N	N	N
L	5.5	P	N	N	N	N	P	N	N	N	P	N	N	H	5.5	N	N	N	N	P	P	P	P	P	P	N	N	U	5.5	N	N	N	N	P	P	P	P	P	P	N	N
L	6.3	P	N	N	N	N	P	N	N	N	P	N	N	H	6.3	N	N	N	N	P	P	N	N	P	P	P	P	U	6.3	N	N	N	N	P	P	P	P	P	P	N	N
L	7.0	N	P	N	N	P	P	P	P	N	N	P	P	H	7.0	N	N	N	N	P	P	N	N	P	P	P	P	U	7.0	P	P	N	N	N	N	N	N	P	N	P	P
L	7.8	P	N	N	N	P	P	P	P	N	P	N	N	H	7.8	P	N	N	N	N	P	P	P	P	P	P	P	U	7.8	P	N	N	N	N	P	P	P	P	P	N	N
L	8.6	P	P	N	N	N	N	P	P	P	P	P	P	H	8.6	P	N	N	N	P	P	P	P	N	P	P	P	U	8.6	N	N	N	N	P	P	P	P	P	P	P	P
L	9.4	N	N	P	P	P	P	P	P	N	N	N	N	H	9.4	P	P	P	P	N	N	P	P	N	P	P	P	U	9.4	N	N	N	N	P	P	P	P	P	P	P	P
L	10.2	P	P	P	P	N	N	P	P	N	N	N	N	H	10.2	P	P	P	P	N	P	N	N	N	N	N	N	U	10.2	N	N	N	N	P	P	P	P	N	P	N	N
L	10.9	P	P	N	N	N	N	P	P	P	P	N	N	H	10.9	P	P	P	P	N	N	N	N	N	N	N	N	U	10.9	N	N	P	P	P	P	P	P	P	P	N	N
L	11.7	N	N	N	N	P	P	P	P	P	N	P	P	H	11.7	N	N	N	N	P	P	P	P	N	P	N	N	U	11.7	N	N	P	P	P	P	N	N	N	N	P	P
L	12.5	P	P	P	P	P	N	N	N	N	N	N	N	H	12.5	P	N	P	P	P	P	N	N	N	P	N	N	U	12.5	N	N	P	P	N	N	N	N	P	P	P	P
L	13.3	N	N	N	N	P	P	N	N	N	P	P	P	H	13.3	N	N	N	N	P	P	P	P	N	P	N	N	U	13.3	P	P	P	P	N	N	N	N	P	N	P	P
L	14.1	N	P	N	N	P	N	N	N	N	N	P	P	H	14.1	N	N	P	P	P	N	P	P	N	P	P	P	U	14.1	P	P	N	N	N	N	P	P	N	N	N	N
L	14.8	P	P	P	P	N	N	N	N	P	P	P	P	H	14.8	P	P	P	P	N	N	P	P	N	N	N	N	U	14.8	P	P	P	P	N	N	N	N	N	N	N	N
L	15.6	N	N	N	N	N	N	N	N	P	P	P	P	H	15.6	P	N	P	P	N	P	N	N	N	N	N	N	U	15.6	P	P	P	P	N	N	N	N	P	N	N	N
L	16.4	P	N	P	P	N	N	P	P	P	P	N	N	H	16.4	P	P	N	N	N	N	P	P	P	P	P	P	U	16.4	N	N	P	P	P	P	N	N	N	N	N	N
L	17.2	N	N	P	P	N	N	P	P	N	P	N	N	H	17.2	P	P	P	P	N	N	P	P	N	N	N	N	U	17.2	P	N	N	N	N	P	N	N	N	P	N	N
L	18.0	N	N	P	P	P	P	P	P	N	P	N	N	H	18.0	P	P	P	P	N	N	N	N	N	N	P	P	U	18.0	P	P	P	P	N	N	P	P	P	P	N	N
L	18.8	N	N	P	P	P	P	P	P	N	P	N	N	H	18.8	P	P	P	P	N	N	N	N	N	P	N	N	U	18.8	P	P	N	N	N	N	P	P	P	N	P	P
L	19.5	P	N	N	N	N	N	P	P	P	P	N	N	H	19.5	P	N	N	N	P	P	P	P	N	N	N	N	U	19.5	N	N	P	P	P	P	P	P	N	P	N	N
L	20.3	N	N	P	P	N	N	P	P	P	P	N	N	H	20.3	N	N	N	N	N	P	N	N	N	P	P	P	U	20.3	P	P	P	P	N	N	N	N	N	N	P	P
L	21.1	N	N	N	N	P	P	P	P	P	P	N	N	H	21.1	P	P	P	P	N	N	P	P	N	P	N	N	U	21.1	P	P	P	P	N	N	N	N	N	P	P	P
L	21.9	N	P	P	P	N	N	N	N	P	N	N	N	H	21.9	P	P	P	P	N	N	P	P	P	P	N	N	U	21.9	P	P	P	P	N	N	N	N	N	N	N	N
L	22.7	N	N	N	N	P	P	P	P	P	P	N	N	H	22.7	P	P	P	P	N	N	N	N	N	N	N	N	U	22.7	P	P	P	P	N	N	N	N	N	N	P	P
L	23.4	N	N	P	P	N	P	P	P	P	P	N	N	H	23.4	P	P	P	P	N	N	N	N	P	P	P	P	U	23.4	P	P	P	P	N	N	N	N	N	N	N	N
L	24.2	N	N	N	N	P	P	P	P	P	P	P	P	H	24.2	P	N	P	P	N	N	N	N	P	P	P	P	U	24.2	P	P	P	P	N	N	P	P	N	N	N	N
L	25.0	P	P	N	N	N	N	P	P	P	P	N	N	H	25.0	P	P	P	P	P	N	P	P	P	N	P	P	U	25.0	P	P	P	P	N	N	N	N	P	N	N	N
L	25.8	P	P	P	P	N	N	P	P	N	N	N	N	H	25.8	P	P	P	P	N	N	P	P	P	N	N	N	U	25.8	P	P	P	P	N	N	P	P	N	N	N	N
L	26.6	P	P	P	P	N	N	N	N	N	N	P	P	H	26.6	P	P	P	P	N	N	P	P	P	P	P	P	U	26.6	P	N	P	P	N	N	N	N	N	P	N	N
L	27.3	P	P	P	P	N	N	N	N	P	P	N	N	H	27.3	P	P	P	P	N	N	P	P	N	N	N	N	U	27.3	P	P	P	P	N	N	P	P	N	N	P	P
L	28.1	P	P	P	P	N	N	P	P	N	N	N	N	H	28.1	N	N	P	P	P	P	P	P	P	P	N	N	U	28.1	N	N	P	P	P	P	N	N	N	P	P	P
L	28.9	P	P	P	P	N	N	N	N	N	P	N	N	H	28.9	P	P	P	P	N	N	P	P	P	N	P	P	U	28.9	P	P	P	P	N	N	N	N	P	N	P	P
L	29.7	P	P	P	P	N	N	N	N	P	N	P	P	H	29.7	P	P	P	P	N	N	N	N	P	P	P	P	U	29.7	P	N	P	P	P	P	P	P	N	N	N	N

In the case of drinking action, the whole measurement data included the dynamics of picking up a cup for drinking and returning to the original rest posture. The change from negative to positive in the case of PD at 4.7 Hz lower arm tremor may suggest a re-emergence of kinetic tremor occurring during the repeated drinking that has been reported in the previous work that no tremor was seen at the beginning of the action, while it was then observed in the later part^13^. However, further study is required to understand and confirm the capture of re-emergent kinetic tremor. The results reported herein serve as the demonstration that the ALW has the potential to provide such information for tremor study.

**Figure 10:**
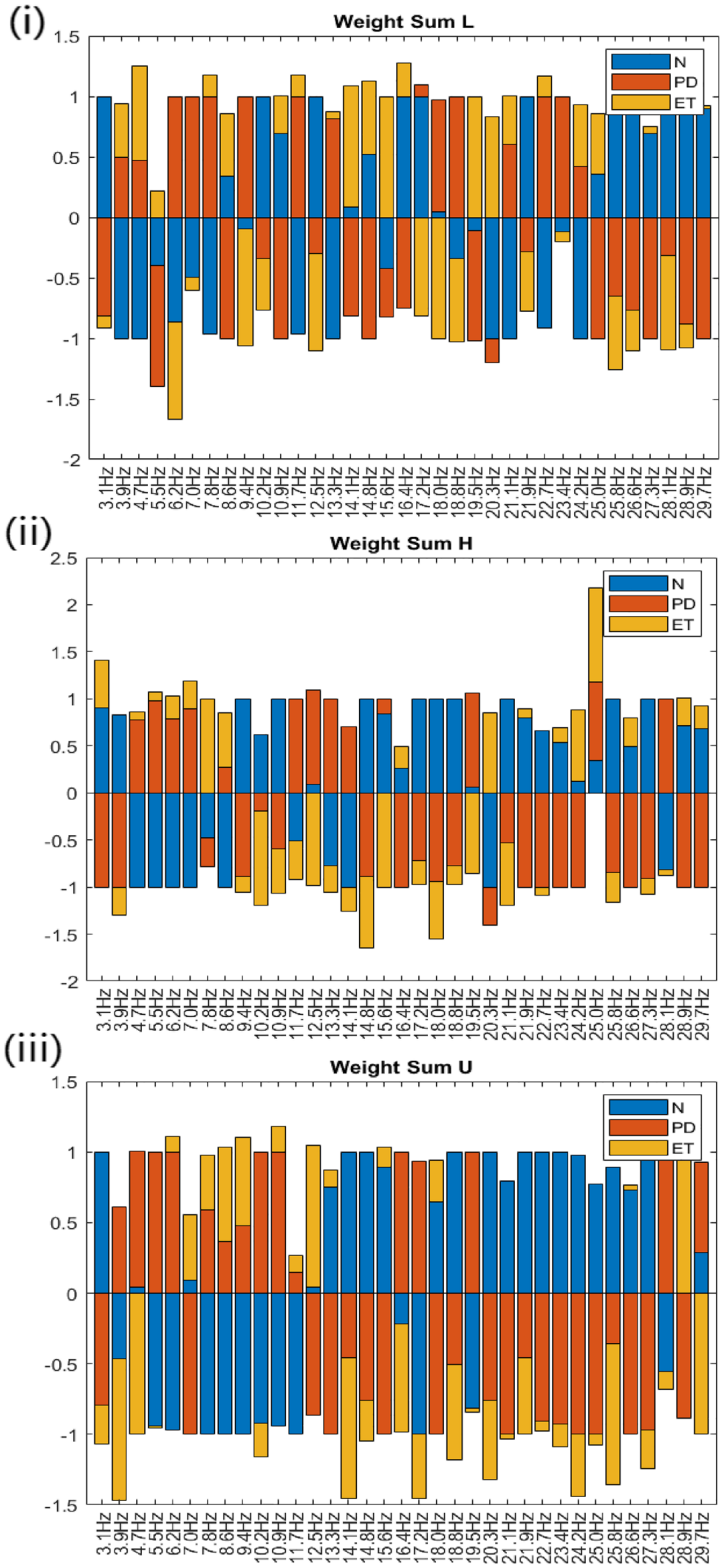
Bar chart of weights summed across all timesteps of each frequency for lower arm, hand and upper arm. The sum of weights are normalised to range (− 1, 1) across classes for better comparison.

Figure 10 shows the bar chart of sum of weights across all timesteps. This is used to signify the total contribution of one frequency towards one class. Despite having some sign changes across timesteps, one frequency might tend to have an overall positive or negative correlation towards one class.

**Figure 11:**
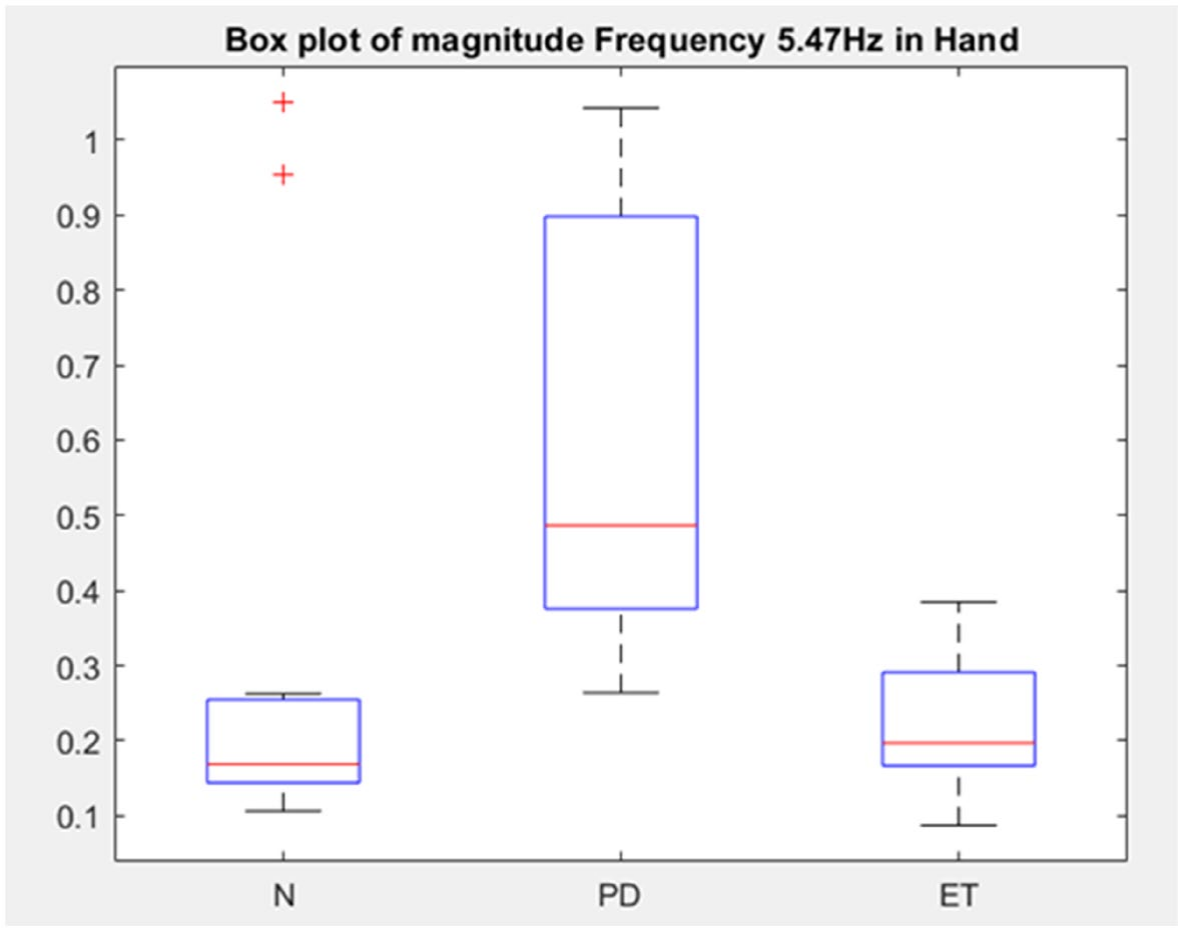
Box plot of average tremor magnitude of frequency 5.47 Hz.

From Fig. 10, differentiating characteristics of N, PD and ET in terms of the relative magnitude of frequency in lower arm, upper arm or hand can be identified. If the weighted sum is positive, the frequency content at the corresponding body part is higher for that class, and the reverse is true if the weighted sum is negative. The features are summarised in Table 5. As a proof of concept, a box plot of magnitude tremor frequency at 5.47 Hz in hand for the datasets is shown in Fig. 11. According to Table 5, the magnitude of tremor at this frequency and body part is higher in PD and ET subjects, and lower in N subjects, which is proven in the box plot, since the mean of PD and ET is higher than the mean of N. The weights give a clue that the magnitude at a frequency is relatively higher or lower, considering all subjects overall but some subjects may have lower values as shown in the box plot. The asterisk (*) marked on the Table 5 are the features that are unique based on the ALW. Note that these features will not classify the data well if used individually. All features must be used for classification with better performance. The applicability of the reported unique features needs further validation with doctor’ judgment.

**Table 5:** Table summarising the differentiating features of N, PD or ET. High denotes that the class has a relatively higher magnitude of frequency content in the limb area compared to the other classes.

Part	f (Hz)	N	PD	ET	Part	f (Hz)	N	PD	ET	Part	f (Hz)	N	PD	ET
L	3.1	High*	Low	Low	H	3.1	High	Low	High	U	3.1	High*	Low	Low
L	3.9	Low	High	High	H	3.9	High*	Low	Low	U	3.9	Low	High*	Low
L	4.7	Low	High	High	H	4.7	Low	High	High	U	4.7	High	High	Low
L	5.5	Low	Low	High	H	5.5	Low	High	High	U	5.5	Low	High	Low
L	6.3	Low	High*	Low	H	6.3	Low	High	High	U	6.3	Low	High	High
L	7.0	Low	High*	Low	H	7.0	Low	High	High	U	7.0	High	Low	High
L	7.8	Low	High	High	H	7.8	Low	Low	High	U	7.8	Low	High	High
L	8.6	High	Low	High	H	8.6	Low	High	High	U	8.6	Low	High	High
L	9.4	Low	High*	Low	H	9.4	High*	Low	Low	U	9.4	Low	High	High
L	10.2	High*	Low	Low	H	10.2	High*	Low	Low	U	10.2	Low	High*	Low
L	10.9	High	Low	High	H	10.9	High*	Low	Low	U	10.9	Low	High	High
L	11.7	Low	High	High	H	11.7	Low	High	Low	U	11.7	Low	High	High
L	12.5	High*	Low	Low	H	12.5	High	High	Low	U	12.5	High	Low	High
L	13.3	Low	High	High	H	13.3	Low	High*	Low	U	13.3	High	Low	High
L	14.1	High	Low	High	H	14.1	Low	High*	Low	U	14.1	High*	Low	Low
L	14.8	High	Low	High	H	14.8	High	Low	Low	U	14.8	High*	Low	Low
L	15.6	Low	Low	High	H	15.6	High	High	Low	U	15.6	High	Low	High
L	16.4	High	Low	High	H	16.4	High	Low	High	U	16.4	Low	High*	Low
L	17.2	High	High	Low	H	17.2	High*	Low	Low	U	17.2	Low	High*	Low
L	18.0	High	High	Low	H	18.0	High*	Low	Low	U	18.0	High	Low	High
L	18.8	Low	High*	Low	H	18.8	High*	Low	Low	U	18.8	High*	Low	Low
L	19.5	Low	Low	High*	H	19.5	High	High	Low	U	19.5	Low	High*	Low
L	20.3	Low	Low	High*	H	20.3	Low	Low	High	U	20.3	High*	Low	Low
L	21.1	Low	High	High	H	21.1	High*	Low	Low	U	21.1	High*	Low	Low
L	21.9	High*	Low	Low	H	21.9	High	Low	High	U	21.9	High*	Low	Low
L	22.7	Low	High	High	H	22.7	High*	Low	Low	U	22.7	High*	Low	Low
L	23.4	Low	High*	Low	H	23.4	High	Low	High	U	23.4	High*	Low	Low
L	24.2	Low	High	High	H	24.2	High	Low	High	U	24.2	High*	Low	Low
L	25.0	High	Low	High	H	25.0	High	High	High	U	25.0	High*	Low	Low
L	25.8	High*	Low	Low	H	25.8	High*	Low	Low	U	25.8	High*	Low	Low
L	26.6	High*	Low	Low	H	26.6	High	Low	High	U	26.6	High	Low	High
L	27.3	High	Low	High	H	27.3	High*	Low	Low	U	27.3	High*	Low	Low
L	28.1	High*	Low	Low	H	28.1	Low	High*	Low	U	28.1	Low	High*	Low
L	28.9	High*	Low	Low	H	28.9	High	Low	High	U	28.9	Low	Low	High*
L	29.7	High	Low	High	H	29.7	High	Low	High	U	29.7	High	High	Low

Low denotes that the class has a relatively lower magnitude of the frequency content. The *denote the case that is unique to the class.

### Comparison with existing medical knowledge

The tremor features are identified from a dataset consisting of 86 PD patients (55 males, 31 females, mean age 69.67 ± 8.18), 18 ET patients (10 males, eight females, mean age 67.06 ± 8.93), and 67 normal subjects (35 males, 32 females, mean age 55.03 ± 10.8).

The characteristics of this classifier can be broken down and inspected parameter-wise. Since drinking action is used, it is likely that the tremor detected is action tremor or kinetic tremor. According to Puschmann et al.^2^, Lenka et al.^14^, and Thenganatt et al.^15^, the frequency of PD’s tremor lies between 4 and 6 Hz. From the identified features for PD, it is found that PD has a high amplitude of frequency at 4.7 Hz in the lower arm, 4.7 Hz, and 5.5 Hz both in the hand and in the upper arm (refer to Table 5). According to Elble^16^, the fundamental frequency of essential tremor lies between 4 and 12 Hz. In the differentiating features identified, ET has high amplitude of frequency at 4.7 Hz to 5.5 Hz, 7.8 Hz to 8.6 Hz, 10.9 Hz to 11.7 Hz in the lower arm; 4.7 Hz to 8.6 Hz in the hand, and 6.3 Hz to 9.4 Hz and 10.9 Hz to 11.3 Hz in the upper arm. This supports the description of ET in Bhidayasiri^17^, which mentions that essential tremor may exist as 4 Hz to 12 Hz action tremors in the upper arm and lower arm.

Aside from matching the current medical knowledge of the frequency content of tremors, the features also introduce a new understanding of the differences between N, PD, and ET in terms of frequency content at the lower arm, hand, and upper arm. The features identified also describe the relative amplitude of frequency content at higher frequencies. The newfound features have the potential to be used in the differential diagnosis of PD and ET since the features clearly define the relative magnitude of frequencies of range 3 to 30 Hz.

Despite most literature discrediting the presence of tremor of frequency higher than 15 Hz, it is also observed that normal patients’ tremor tends to have a relatively higher amplitude of frequency at 20–30 Hz at all body parts. This is suspected to be a physiological tremor. Since the average magnitudes of these frequencies are extremely low, they are possibly invisible, undetected, and negligible during raw measurement.

Normal patients also have a relatively high amplitude of frequency at 3.1 Hz, which suggests voluntary drinking motion. It is highly likely that PD and ET have lower amplitudes at 3.1 Hz because they are overshadowed by high amplitudes of frequency at 4 Hz to 6 Hz.

The frequency magnitude might be different for each body part. However, the relative amplitudes in some frequencies are consistently high for the lower arm, hand, and upper arm. This includes 3.1 Hz, 12.5 Hz, 18 Hz, 21.9 Hz, 25–27.3 Hz, 29.7 Hz for N; 4.7 Hz, 6.2 Hz and 11.7 Hz for PD; and 7.8–8.6 Hz for ET (refer to Table 5).

The features identified are consistent with the current medical knowledge to some degree. The inconsistencies might be caused by inaccuracies of the neural network, which has only achieved 80% accuracy, and the inaccuracies caused by the linear weights approximation, which has 73% accuracy. The limited datasets used in the training are one of the causes since oversampling is used to upsize the ET data. Thus, it is highly possible that overfitting has occurred in training. The input feature used is STFT, which captures stationary and linear data, which is deemed by some literature to be an unfeasible option for feature extraction since tremor is non-stationary and non-linear^18^. HHT is recommended instead; however, the neural networks trained using HHT as the input features have achieved low performance. Besides, the data is recorded during drinking action, which causes dynamic action tremors, which might introduce noises in the data. All of these factors cause inaccuracies in the differentiating features identified. In future studies, the work can be further tested on any potential biases and through validation with clinicians’ judgment.

The work shows that the ALW, which is the linear simplification of the neural network, is useful in simplifying the neural network for faster computation as the neural network is reduced to one single layer. Since ALW is branching off from LRP and has its own classification algorithm, ALW can be used to evaluate and validate the observations from LRP.

The simplification of LRP outcome with ALW comes at the expense of lower accuracy in the classification. The main limitation of the LRP-based ALW method is that the non-linearity of the neural network is neglected. Nevertheless, the demonstration of ALW to be interpreted easily, particularly through the results in Table 5 on the cardinal information in distinguishing the tremors, indicates the better explainability of ALW. There is a demand for approaches that have the capability to explain the decision-making process for the adoption of AI in healthcare^19^.

## Conclusion

An RNN is designed and trained to differentiate between N, PD, and ET, and an accuracy of 80% is achieved. Differentiating features between N, PD, and ET are identified in terms of magnitudes of frequency content in the tremors. Using these features in ALW, an accuracy of 73% is achieved.

The LRP-based ALW method is able to simplify the internal workings of neural networks into a single 2D convolutional layer for interpretation purposes. Using this method, the clues on the unique symphony of frequency contents of tremors of normal subjects, PD patients, and ET patients were found. The future study will cater for the training with larger samples and different subgroups of PD and ET to improve the overall classification capability and the generalisability of the explained characteristics.

### Supplementary Information

Supplementary Information.

## Data Availability

The dataset used and/or analysed during the current study available from the corresponding author on reasonable request.
