# Supplementary material for: Explainable artificial intelligence for searching frequency characteristics in Parkinson’s disease tremor
Source: Sci Rep. 2023 Oct 30;13:18622. doi: 10.1038/s41598-023-45802-z (PMC10616175; doi:10.1038/s41598-023-45802-z)
Supplement: Supplementary file 1 — Supplementary Information. [file 41598_2023_45802_MOESM1_ESM.pdf]

Appendix

Explainable artificial intelligence for searching frequency characteristics in Parkinson's disease tremor

The ALWs are based on the neural network trained on input parameters derived from STFT simplified and drinking action. The results is as shown in Supplementary figures 1. Some of the general signs of one class are unique to the other classes. For example, for frequency of 3.12Hz at lower arm (Supplementary figure 2), ALW for N is positive, while the ALW for PD and ET are negative. This describes differentiating features for N, since it stands out from the other classes.

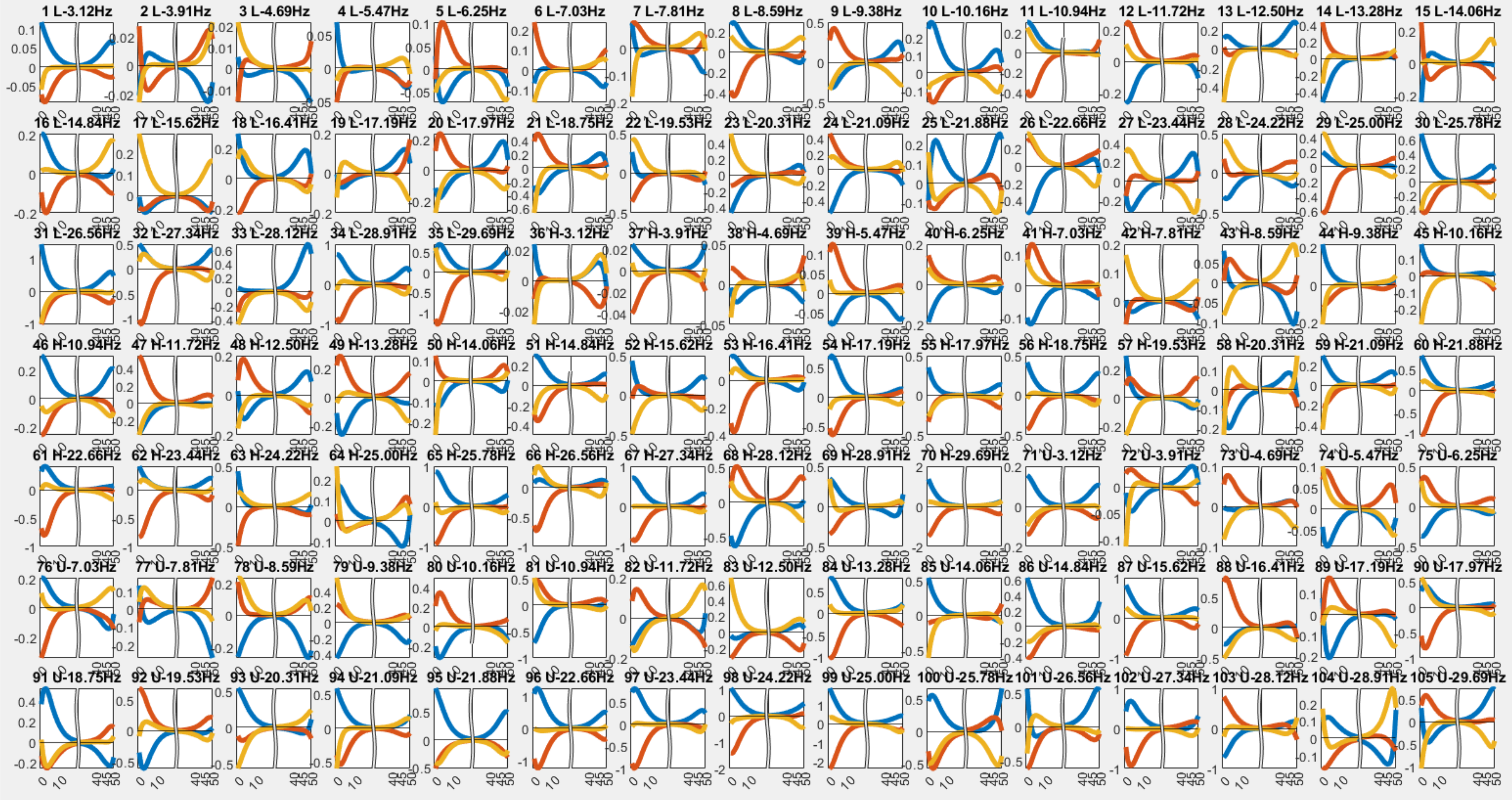

Supplementary Figure 1: Visualisation of the ALWs as a function of time steps for N (Blue), PD (Red) and ET (Yellow). The title is in the format of 'P-f' where P is the body part where the tremor is measured, f is the frequency that the input parameter corresponds to.

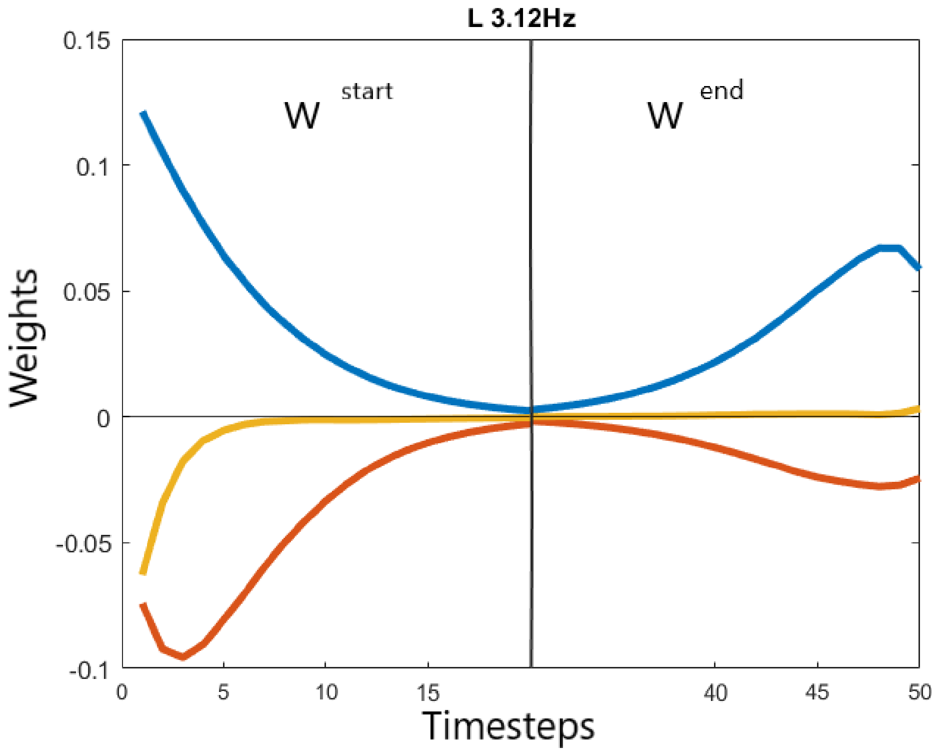

Supplementary Figure 2: Zoomed-in graph of one of the subplots in Supplementary Figure 1, completed with region labels, x-axis label and y-axis label.
